# Struggles over elder care in South Africa

**DOI:** 10.1080/0376835X.2024.2374823

**Published:** 2024-08-09

**Authors:** Elena Moore, Gabrielle Kelly

**Affiliations:** Department of Sociology, Leslie Social Science Building, University of Cape Town, Rondebosch, South Africa

**Keywords:** Elder care, South Africa, care, careworkers, social protection, community care

## Abstract

This paper examines the tensions and struggles over elder care that are happening in South Africa between family caregivers, care workers and the state. These struggles are experienced and expressed at the familial and community level but are best understood by examining how the state has de-funded facilities in favour of ‘ageing in place’ without any additional investment in community and home-based care support. The findings reveal that stresses on unpaid and paid carers are expressed in terms of individual failings and interpersonal conflict rather than through a socio-political contextual lens. These findings contribute to how a familialist care regime impacts the everyday personal relationships between paid and unpaid carers of older persons.

… it’s very sad some people appreciate it, and some don’t. Some want to shout at you on the street ‘like nurse, doctor, pharmacist but I can’t do diagnostics. People need their tablets … When you get to the person’s house with the tablets and do chronic adherence, people take advantage of you, as if they are paying you to do a service for them … .

Miriam, a care worker^1^, described her experience of providing elderly care in her community. She expressed the expectation for her to go beyond her responsibilities, as a contract home-based carer responsible for neonatal care, TB/HIV medication adherence and the care of older persons. Sitting alongside such accounts are perceptions and experiences shared by family caregivers who report that social workers and home-based carers are not providing a decent service. Family caregivers expressed the difficulty in accessing home-based care support both in terms of receiving the service but also in terms of the quality of care they provide, as one family caregiver stated: ‘home care is provided by demotivated, poorly paid people who don’t clean, eat people’s food and are not held accountable, and it is not clear how quality assurance processes work.’ This article shows how stresses to unpaid and paid care for older persons are expressed in terms of personal failings and interpersonal conflict, rather than through a socio-political contextual lens.

Older persons and carers struggle to provide and receive care under the current economic conditions. We argue that despite a dominant policy emphasis on ‘ageing in place’^2^ there hasn’t been the necessary public funding to support it. More importantly, our findings reveal that funding for community care of older persons has shrunk over the last 18 years and the financial cuts imposed on elderly care trickle down and impact care relations. The funding cuts are located in a context of dire socio-economic crises, with high levels of unemployment, poverty and increasing elderly care needs. With the shrinking budget allocations for community care, NPOs, as service providers, are expected to do more with less. Decreasing funding in the elderly care sector not only impacts the relations between careworkers and older persons, but it ultimately impacts the quality of care. The cuts to community care mean the care falls to female family caregivers.

In this article, we will present an overview of the changes in elder care in post-apartheid South Africa. The aim of the paper is twofold. First, it sketches the major developments of national policy on elderly care, set in the wider context of social policy and we argue that the reduction in spending in community care indicates a deepening familialist approach to family and care policies in South Africa. We will set out how policy developments for older persons in South Africa have followed the prevailing regime for welfare and care that relies heavily on social grants but fails to meet care needs. Secondly, and more specifically, the paper reveals how the underinvestment in community care at a time of increased need for direct care provision in the context of population ageing can shape growing tensions between paid and unpaid care workers.

## 1. Elderly care post-apartheid

The historical legacies of apartheid’s care infrastructure for direct care provision for older persons continues to shape the contemporary landscape. Services and support for most coloured, black and Indian older persons in South Africa during the apartheid era were virtually non-existent (Sagner & Mtati, 1999). In 1928, the state introduced the old-age pension for white and coloured persons which was later, in 1944 broadened to include African and Indian persons. Like much of the social welfare of the time, the state pension was discriminated along racial lines both in eligibility and benefit levels (Sagner, 2000). Sagner (2000:528) stated that from ‘the very start the state pension system was conceptualised as an incentive to induce people to care for dependent relatives rather than as a fully fledged social programme that would guarantee economic security for the aged.’ Sagner (2000) explained how benefit levels were purposefully kept low, to require and expect adult children to support their parents.

The South African welfare and care regime for most of the twentieth century was thoroughly dichotomised (Seekings & Moore, 2014). Whereas less advantaged white South Africans were given much assistance, very little support was allocated to the majority African population. Sagner (2000:539) demonstrated that throughout the 50s, 60s and 70s governments sought to cut down on the value of African state pensioners as discrimination between the races became greater in the period 1948–60. In the 1970s, the apartheid state began to reduce racial discrimination in benefits and finally established parity in 1993, on the eve of the country’s first democratic elections (in 1994). Whilst apartheid welfare policies were deeply racialised and discriminatory, the National Party were also deeply conservative and was largely opposed to the idea of a ‘welfare state’ (Seekings, 2020:1159). Seekings revealed how the National Party was slow to introduce public provisions for white citizens during apartheid and ‘left almost all contributory welfare programmes to the private sector’ (Seekings, 2020:1159). White individuals were expected to take responsibility for themselves and their own elderly care by buying into private pension schemes, which was made easier through their preferential education and employment positions. In the final decades of apartheid marketisation was driven by the then government who supported the introduction of markets in the provision of elder care.

The post-apartheid state deracialised the provision of care (Button et al., 2018:604). Whilst pension roll out was deracialised, the state considered the value in extending residential care homes but argued that ‘it was inappropriate on both ideological factors as well as fiscal constraints’ (Author, 2014:427) and opted to desegregate existing institutions without increasing expenditure in communities that had been under-served for decades.

## 2. State familial policies in welfare and family policy in South Africa

Sevenhuijsen et al. (2003:312) argued that South Africa draws ‘heavily on a familialist model of care.’ Through an assessment of guiding policy frameworks, they argued that care is located in the separate private sphere without a related consideration of the situation in which families find themselves and that family life is described in ‘gender-neutral, functionalist and moral terms’ (2003:306). Scholars have repeatedly contended that the familialist ethos (Sunde & Bozalek, 1995) is entrenched in policies, initiated by apartheid policies, compounded by the African philosophy of communitarianism (Gouws & Van Zyl, 2015) and deepened by a discourse of self-reliance in the post-apartheid policy frameworks (Bond, 2014).

In our review, we considered how a familialist approach entrenched in policies is implemented in practice through a review of spending on eldercare. Drawing on Saraceno’s (2016) five policy types and varieties of familialism, South African’s elderly care policy approach on paper can be defined as a ‘supported familialist approach.’ Supported familism exists when policies support caregivers. With this approach, as we will outline below, there is some provision for community care (residential care for very few, home-based care for some) and limited cash transfers (Grant-in-Aid) to contribute to the cost of caregiving for fully dependent older persons. However supported familialist policies when unsupported by public funds, shifts South African policies more closely towards a ‘familialism by default’ approach. A familialism by default approach refers to a policy context where the state doesn’t offer publicly provided support and assumes the family will care. The familialist by default approach becomes more visible in the absence of budgetary commitments to supporting family and community care for older persons. Whilst policies may explicitly endorse a supported familialist approach, in the absence of adequate funding for service centres, home-based carers and residential care we see how the state views families as responsible for the welfare of their members through budget allocations. We argue, and what we show through our findings, the post-apartheid state has retreated deeper into a familialism by default approach to elderly care.

## 3. Older person policy priorities and state spending

The generosity and legacy of the state pension, in the absence of direct care provision must not blind one to the fact that the focus on grants is inherently familialist, i.e. it emphasises (economic) self-reliance and independence for elderly people. The state has positioned itself as ‘facilitating’ and ‘supporting’ caregiving in families, rather than meeting care needs directly (Sevenhuijsen et al., 2003:203; Button et al., 2018:605). The state supports families in caring for the elderly and provides for the needs of the elderly through the Older Persons Grant (hereafter referred to as the OPG) and a lesser extent the Grant in Aid (hereafter referred to the GIA). The OPG is means-tested and 73.1 per cent of older persons, representing 3.8 million persons aged 60 years and older received a social grant in 2022. Men and women are eligible from the age of 60. The OPG in 2023 was valued at R2080 per month. Approximately two of three OPGs are paid to women and the value of the grant is significantly higher than the value of child support grants. The OPG is not assessed in terms of actual need, i.e. the care needs of the elderly and a guarantee of economic security for older persons. The state relies heavily on the care work of families and female caregivers to ensure the care needs of older persons are met.

The GIA social grant is available to older persons who have full-time care needs. The older person is assessed by a medical professional as in need of ‘regular attendance by another person’ and it subsidises care needs for those who are not in residential care facilities. In 2022, 252 161 recipients were receiving R505 at a cost of R121 million. Only 6.5 per cent of OPG beneficiaries receive the GIA.

The formal elderly care services that exist in South Africa include residential care homes and service centres. There are 394 subsidised residential care homes catering to 18 011 older persons. The geographical spread of residential homes is uneven and almost one-third of the residential care beds are located in the Western Province. On the other hand, there are 78 477 older persons receiving support from service centres which are provided largely by NPOs with inadequate funding. Services are unevenly distributed across the country and only cater to older persons who can access the centre. They generally focus on active older adults through meal provision, social clubs and sometimes-primary healthcare services. As will be revealed below, there are no home-based or community-based care workers specifically addressing geriatric care or the care needs of older persons. Community-based care workers and home-based carers located in communities are responsible for a range of patients, including TB/HIV medication adherence, and neo-natal care and are not able to provide much support to older persons. It should be noted that care workers can be supported by the state via the Social Employment Fund, the Extended Public Works Programme, the Department of Health, the Department of Social Development or through private funding raised by non-profit organisations. Most careworkers working in the area of geriatric care have extremely limited training.

In the table below, the ‘combined spending on Older Persons Programme provincially’ is the total amount paid towards community care, i.e. subsidies to residential care and service centres.

The Older Persons Act (2006), which provides a regulatory framework for the provision of care and support to older persons, emphasises family – and community-based care. One of the main objectives of the Act, outlined in section 2 (c) is to: ‘ … shift the emphasis from institutional care to community-based care in order to ensure that an older person remains in his or her home within the community for as long as possible.’ Given the priority assigned to community and family-based care, we analysed the spending on community care and family-based care services for older persons in South Africa which involved a review of national older persons programming and budgets in national and provincial Annual reports, Annual performance plans, and Estimates of Provincial Revenue and Expenditure, and Budget Statements for Department of Social Development (hereafter referred to as DSD) from 2006/2007 to 2023/2024, as well as engagement with NPOs organisations receiving funding for elder care services (Moore, 2023b).^3^

Most funding to the older persons sector is provided by DSD. This spending for the 2006/2007 to the 2023/2024 period is outlined in Table 1 below. Whilst the economic value of the support to older persons in South Africa has grown, it has not been increased sufficiently to meet the needs of a growing older population, nor has it been adequately linked to inflation. The older person population has grown from 3.4 million in 2006–5.6 million in 2022. The spending per person adjusted for inflation is 5 per cent higher in 2022/2023 than in 2006/2007, but funding to provinces, which are responsible for care provision, is down 13 per cent since 2006/2007 (Moore & Kelly, 2023b). State spending is not prioritising community care despite the policy focus on supporting community- and family-based care.

**Table 1.** Department of social development spending on older persons (2006/2007 - 2022/2023).

Spending type	2006/2007	2013/2014	2019/2020	2020/2021	2021/2022	2022/2023 adjusted appropriation
OPG spending	21 861 890 000	44 064 239 000	83 488 248 000	81 024 952 000	84 102 284 000	92 125 465 000
OPG numbers	2 200 000	3 000 000	3 700 000	3 700 000	3 800 000	3 800 000
**GIA spending	–	–	–	–	–	1 482 678 600
GIA numbers	–	–	–	–	–	252 161
Older Persons Policy Development/Support	4 654 000	22 371 000	18 000 000	9 400 000	12 000 000	19 300 000
Total national DSD	21 866 544 000	44 086 610 000	83 506 248 000	81 034 352 000	84 114 284 000	92 144 765 000
Combined spending on Older Persons Programme provincially (9 provinces)	460 139 888	917 721 000	1 510 475 000	1504810000	1573331274	1 606 383 000
Total National and Provincial DSD spending combined	22 326 683 888	45 004 331 000	85 016 723 000	82 539 162 000	85 687 615 274	95 233 826 600
						
Spending in 2022/2023 terms	50 993 508 441	66 404 602 875	93 331 549 081	87 962 491 911	87 661 949 755	92 125 465 000
2022/23 vs year in question	81%	39%	−1%	5%	5%	
**Per person (OPG)**	23 179	22 135	25 225	23 774	23 069	24 244
2022/23 vs year in question	5%	10%	−4%	2%	5%	
						
Older Persons Policy Development/Support	10 855 593	33 712 993	20 122 208	10 204 849	12 507 905	19 300 000
Total national DSD	51 004 364 035	66 438 315 868	93 351 671 289	87 972 696 761	87 674 457 660	92 144 765 000
Combined spending on Older Persons Programme provincially (9 provinces)	1 073 289 970	1 383 001 271	1 688 560 665	1 633 655 241	1 639 923 205	1 606 383 000
Total National and Provincial DSD spending combined	52 077 654 005	67 821 317 139	95 040 231 954	89 606 352 001	89 314 380 865	95 233 826 600
**Change**	**2006/2007**	**2013/2014**	**2019/2020**	**2020/2021**	**2021/2022**	**2022/2023 adjusted appropriation**
Older Persons Policy Development/Support	78%	−43%	−4%	89%	54%	
Total national DSD	81%	39%	−1%	5%	5%	
Combined spending on Older Persons Programme provincially (9 provinces)	50%	16%	−5%	−2%	−2%	
Total National and Provincial DSD spending combined	83%	40%	0%	6%	7%	
**Per person (OPG)**	**2006/2007**	**2013/2014**	**2019/2020**	**2020/2021**	**2021/2022**	**2022/2023 adjusted appropriation**
Older Persons Policy Development/Support	4,9	15,3	9,1	4,6	5,7	8,8
Total national DSD	23 183,8	30 199,2	42 432,6	39 987,6	39 852,0	41 884,0
Combined spending on Older Persons Programme provincially (9 provinces)	487,9	628,6	767,5	742,6	745,4	730,2
Total National and Provincial DSD spending combined	23 671,7	30 827,9	43 200,1	40 730,2	40 597,4	43 288,1
**Change**	**2006/2007**	**2013/2014**	**2019/2020**	**2020/2021**	**2021/2022**	**2022/2023 adjusted appropriation**
Older Persons Policy Development/Support	78%	−43%	−4%	89%	54%	
Total national DSD	81%	39%	−1%	5%	5%	
Combined spending on Older Persons Programme provincially (9 provinces)	50%	16%	−5%	−2%	−2%	
Total National and Provincial DSD spending combined	83%	40%	0%	6%	7%	

In 2022/2023, the total DSD budget spent for older persons was just over ZAR93.89 billion,5 per cent more in real terms than in 2006/2007. Whilst national funding (which includes budgeting for social grants, national policy development and funding to national older persons NPOs), is up 5 per cent, provincial funding is down 13 per cent, so combined national and provincial support from DSD in 2022/23 is up 4 per cent. Looking at 18 years, we also see that funding hasn’t returned to pre-Covid amounts both in terms of total levels and adjusted per person.

Social grant spending represents approximately 98 per cent of all DSD’s spending on older persons. The rise in the number of older persons has led to an increase in the economic cost of the older persons’ grant. The remaining 2 per cent of funding is spent on programme and policy support and development for ZAR19 million in 2022/2023 in the national budget as well as subsidies and transfers paid to NPOs that provide residential care or community-based services to older people at a cost of just over ZAR1.5 billion.

As we argue in this paper, despite state policy on older care prioritising ‘ageing in place’, there is very little funding allocated to NPOs that provide services to older people and their caregivers at the community level. Funding has also decreased over time. Essentially the focus of state support for older persons is on social grants and subsidising the cost of care to the very frail and the very poor. But South Africa has one of the largest, older and fastest-growing populations of older persons in the African region (around 5.6 million or 9 per cent of the population in 2022). This means that there is a decrease in spending at the community level at a time when the older person population is growing, leaving large proportions of the older person population receiving little, if any, community-based care.

## 4. Increase in social care need for older persons

South Africa has a well-established set of laws, policies and programmes for older people across various sectors. However, the scale of inequality, the absolute size of the older population, poor budgetary allocation to the older persons sector, and challenges with governance result in poor implementation (Solanki et al., 2021). Older persons are more vulnerable to poverty and struggle more to exit poverty than their younger counterparts. In South Africa, 62 per cent of persons aged 60 or older are described as black African and are highly likely to have been disadvantaged by the apartheid system during their youth, particularly in terms of access to education and work opportunities (Statistics South Africa, 2023). The OPG is a critical poverty alleviation mechanism, but in the context of high unemployment older persons are often financially supporting multiple family members in multi-generational households with their OPGs (Burns et al., 2005; Schatz & Ogunmefun, 2007). Fifty-two per cent of older people in South Africa live in households with no employed household members. In South Africa, men and women who reach age 60 are likely to live 17- and 20-years additional years respectively (World Health Organization GHO, 2019). However, older people commonly suffer from multiple health conditions, particularly those who are socioeconomically disadvantaged (Barnett et al., 2012). Non-communicable diseases are becoming an increasing contributor to Disability Adjusted Life Years (DALYs) among both the general population and those aged 60 plus (76 per cent of all disability life years in the country are NCD-related). High rates of diabetes (23 per cent) and hypertension (68 per cent) within the older population (Statistics South Africa, 2023) bring with it both an increased need for health care and social care services and increased risk of disability later in life caused by diabetic amputations and strokes if these conditions are not well managed. Managing these conditions and disabilities among older persons happens within families and communities.

Healthy Life Expectancy data show that people in SA living beyond 60 live 5–6 of their remaining years in ill health (Global Health Observatory, WHO). Only 23 per cent of older persons (5.4 per cent of black South Africans) are covered by a medical aid or medical benefit scheme and the majority make use of public clinics and hospitals. Population ageing therefore has significant implications for the already over-burdened and under-resourced health system, which already struggles to provide care to older persons that is accessible, responsive and of sufficient quality (Kelly et al., 2019). It also has significant racialised and gendered consequences as data on healthy life expectancy show us that women not only live longer but are likely to spend more years in poor health and disability with lower quality of life and higher levels of dependence (He et al., 2020). Moreover, women are not only going to require care for longer, but the additional gender dimension is that it will be women who overwhelmingly provide the care.

Declining health and function in old age also has implications for formal and family care systems. Estimates on the number of older persons living in community settings in South Africa who need assistance with at least one Basic Activity of Daily Living (BADL) vary from in one rural area (Harling et al., 2020) to between 38 and 49 per cent of those aged 65–74 and 75 + , respectively (WHO, 2015). One study estimated around 88 per cent of South Africans aged 50 + had ADL difficulties, when instrumental criteria (i.e. Instrumental Activities of Daily Living) such as catching public transport, concentrating and remembering things, or learning a new task, were included (Yaya et al., 2020). Whilst the precise care need for older persons with different conditions in South Africa is unknown, care need increases with age, and the WHO outlined that from age 70, care need increases (WHO, 2015). Lloyd-Sherlock (2019:152) shows that the number of South Africans living beyond 70 is increasing and is set to double by 2040 and reach 3.5 million. Lloyd-Sherlock (2019:154) reminds us of racial disparities in care needs indicating that ‘lifetime disadvantage and exposure to HIV-infections have led to higher rates of dependency and cognitive impairment among older black South Africans.’ It is in this context of an increase in care needs that we examine the state’s role in the provision of care and how it influences care work in families and communities.

With extremely limited state provision of elderly care with less than 394 subsidised residential facilities available only to the frailest and only 1713 registered community-based care and support services, most of which provide socialising and meal opportunities rather than care as the financing structures of community care do not make adequate budget provision for care services to enable NPOs to be able to provide respite to persons with ADL restrictions. With limited economic resources to buy care from the market, older persons requiring care rely on family members and, in some cases home-based care visits provided by community health workers in cases of acute medical need. It is in this context of an increase in care needs that we examine the state’s role in the provision of care and how it influences care work in families and communities.

## 5. Methods

Our evidence is based on two interrelated studies of intergenerational relationships of care in South Africa. The first study examined intergenerational relationships of care, including care for older persons, in multigenerational households in Cape Town and Johannesburg in 2018 and 2019 (Moore, 2023a). Undertaken by the first author, the study interviewed co-residential female employed family caregivers experience of care for an older person in 50 households across the two sites. Sampling requirements included that participants were part of a multigenerational household, and that the caregiver was living in the same household as the older person (a ‘co-resident caregiver’) and in paid employment. Informed consent was obtained from participants at multiple stages of the research. Participants were recruited through formal networks, including health clinics located in middle-class and low-to middle-class suburbs, and from a range of other clubs, such as running and soccer clubs. This strategy was supplemented by snowball sampling (introductions by participants to their colleagues, friends, and neighbours) and looked specifically at the provision of care to older people as experienced by employed family caregivers.

Interviews were conducted with the caregivers. When speaking about care responsibilities for older persons, participants identified themselves as the primary caregiver, understood in terms of the responsibility to provide care themselves or with the support of others. The study sought to explore a range of experiences among women with different incomes and occupations. A total of 20 participants lived in Cape Town and 30 participants lived in Johannesburg; 35 identified as black, eight as white and seven as coloured.

The second study, funded by a Wellcome Trust Award, examines family caregiving of older persons in South(ern) Africa. Whilst research is still ongoing with this work, this paper picks up on the conversations shared at older persons’ community meetings in six sites across the Western Cape and KwaZulu Natal in South Africa. Overall, 120 participants attended the six meetings. At the meetings, there was a mix of family caregivers older persons as care receivers, home-based carers, social workers and other interested community members, such as representatives of senior centres and programmes, as well as members of the Department of Social Development. The meetings were co-hosted and facilitated by partnering organisation that provide services for older persons. In three of the six areas there were no senior service centres, and the meeting was co-facilitated with community members who are active in supporting older persons.

The discussions were held in Afrikaans, English, isiXhosa and isiZulu, depending on the main language of the community in which the meeting took place. Each meeting lasted approximately 90–120 min. The meetings were lightly structured around four discussion points and were led by the participants. The four discussion points focussed on (1) experiences of family caregiving for older persons (2) challenges of family caregiving for older persons in the community (3) services available that support family caregiving (4) support services needed to enable better care for older persons. In the meeting, ideas were captured on A1 posters and extensive notes were taken and shared with the partnering organisation and community members.

The first and second study included the voice of caregivers, who, in both studies, emphasised the need for, but lack of, community support in managing elderly care. The second study, whilst including the voices of older persons and caregivers, heard the experiences of care workers, a critical perspective for understanding the challenges to the provision of community care for older persons. The community meeting method was useful as it allowed different social actors involved in elderly care to discuss experiences and challenges in context. For many older persons, the meeting was the first time they met with care workers, social workers or family caregivers outside of private households. Through the discussion it became apparent that many issues were structural and common to all older persons, i.e. it became apparent that some of the challenges in receiving care were not individualised to a specific social worker or home-based carer or family member. The space allowed each actor to listen to the experiences of each other and have a better understanding of the broader systemic challenges where individual experiences could be better understood. A two-stage thematic analysis of the discussions was undertaken. Community-level analyses were used to get a better understanding of experiences, challenges and support structures in each community. Following this, a comparative analysis was conducted to compare the experiences and challenges across the six sites.

## 6. Care work, older persons and conflict

The tensions expressed by caregivers in caring for older persons will be presented in two ways. Firstly, we will examine the conflicts expressed by paid careworkers who care for older persons in the community. Secondly, we will examine the tensions expressed by unpaid family caregivers in relation to care work within their families and communities. In the second part of the findings, we examine the tensions that arise due to the ways in which care work for older persons is gendered, costly and unsupported by public support.

### 6.1. Care work and conflict within communities

Findings from the community meeting highlighted a failing family discourse within care workers’ perspectives, including older persons as victims. Care workers asserted ideas of failing norms as the source of vulnerability amongst older persons expressing issues around caregivers’ intentions, elder abuse, and neglect. Across all sites, care workers identified younger generations as irresponsible and careless in their responsibility towards older persons and in doing so tended to individualise family failings and overlook the socio-economic setting in which family care occurred, effectively erasing the conditions of places in which care occurs. One care worker stated:

Drug abuse and alcohol abuse in the house. Father has a stroke and then the mother is the caregiver but the real person giving the problems is the drug person  …  The police can’t do anything. Social workers don’t provide support for the elderly.

For many home-based carers it is the complete lack of support from other services that leaves them blaming individual families and family members rather than the state and the lack of services for older persons. Careworkers are unable to help the older persons as the concerns of the older persons and the communities in which they are located are much broader than attending to a particular care need. They use the individual circumstances of the younger generation as an explanation of ‘the family’ being abusive, whilst they are unable to assist the caregiver or older person as their remit is limited. Caregivers can only make referrals to other social services, which they know are limited and in reality, won’t assist. As one care worker explained: ‘Older persons talk about social problems – all we can do is refer. If there is a social problem – welfare. Illness – clinic. Wound they can’t do – clinic.’

Blaming family members takes attention off the lack of services and state support for families and takes attention away from the reality that the older person may be the only person in the household receiving state social grants.

Many caregivers and community members emphasised how younger generations ‘abuse’ the social grants received by the older persons. Community members viewed household relations in this regard as abusive. Some careworkers claimed the social grants were not used to provide decent nourishment for an older person. A repeated concern was that older persons were unable to take medication without making sure they had had food and they stated: ‘sometimes we bring food for the patients because they can’t eat medication without food.’ Service centres are a critical source of food provision for older persons, but with the closure of many service centres during COVID-19, coupled with the financial viability of remaining services centres, many older persons have fewer options for community-based food provision.

In situations where family members are employed, care workers reported that: ‘There are elderly people that they are locked inside. People are working or don’t care. Sometimes grandchildren look after them.’ This points to the restrictions on caregivers and how they balance work and caregiving responsibilities. Employment is prioritised in situations where there are few resources. It is important to consider how these discourses support the design of specific familialist policies that we see in the South Africa White Paper (2021), policies that expect family members to care for older persons with little support.

### 6.2. Care work and conflict within families

In this part of the paper, we consider what it means for family care when there are few options other than family care, i.e. where there is no respite care, or when there are not enough residential care beds and entry requirements are very strict in terms of both frailty and income. What becomes clear through the findings, is that there are people who cannot be properly cared for by family (because of medical complexity or household situation) but must remain in households, and we see how this can create tensions within families.

In considering the tensions about care work of older persons within families, three main issues arise, the first is the gendered, privatised nature of the responsibility that families and largely women carry for care work. Care work of older persons for waged workers remains a private responsibility and women’s responsibility. The first area of conflict arises due to women’s productive and reproductive work colliding in ways where little effort has been made to reconcile the two in South Africa. Though the number of adults caring for their parents has greatly increased, employers have made no provisions to help workers carry out this task. Particularly overworked are family caregivers who are caring simultaneously for wider kin members, including children at the same time as caring for older parents, uncles and aunties. Seeking support from other family members is not always helpful and can cause great tension for employed caregivers who are juggling demands, as Sunki, a 48-year-old female employed family caregiver stated:

I’ve been in a situation where I’ve asked for a family member to come and help because I was out for work and there’s a lot of my family members who are unemployed, but no one came. I ended up paying people and their excuses was I stay far, and it is just two taxis away, ‘no, you stay far’ and it has been a story always.

Disputes can arise when relations between adult children and family members is fractured. Some women in the sample felt that they had little choice but to take on the responsibility.

In addition to long days of work, the additional private responsibility for covering all the expenses is taxing. The level of responsibility that women are carrying causes tensions amongst siblings, as caregivers may seek more support. As Zandile noted:

… you know honestly, I’m not angry or upset about it but at first, I used to say they are older and they are supposed to look after my mom then I said it’s not going to help me and I won’t have a relationship with them but instead they will make me angry

It is also important to stress that most elderly people and families cannot afford hiring care-workers or paying for services matching their real need. This is particularly true of elderly people with illnesses who require day-long care. As a result, covering the costs of reproductive work was the second major source of conflict experienced within families. There were concerns that older persons with particular health conditions were not getting nutritional meals fit for their particular illness, such as diabetes or high blood pressure as other family members spent the OPG without due consideration for dietary needs:

They were buying 10 breads and they store them in fridge then she will warm the bread. She was eating the bread the whole month and mind you this person is diabetic. I kept on asking as to why my aunt had diarrhoea, so my uncle felt that I was after him.

Family members were trying to minimise their expenses and stretch limited social grant resources whilst trying to ensure quality care. With service centres closing and food costs rising, many of the disputes involved conflict over food costs and responsibility for the provision of quality meals for the older person. The context sets the conditions for conflict, which pit family members against one another as they try to control and maximise the little available.

Conflicts also arose as family caregivers felt unsupported by community service providers in getting help with care. Many caregivers spoke about the inability of services to cater for older persons who have mobility challenges and are unable to get to clinics or hospitals. Many spoke about the need for more health care workers to do more home visits, especially when distances and costs of travel are high:

We really have a problem as older people. When I visit some older people [in their houses], they complain that their feet hurt and are swollen Such things can be helped by healthcare workers but there are no healthcare workers available/deployed.

Caregivers highlighted how care workers are not available in under-resourced areas. The community meeting provided a space where people considered the different reasons why social workers are ‘absent.’ Health professionals working in the community explained how social workers cover many areas with large populations and a very wide mandate (including foster care cases which take considerable time) and therefore they have a high and varied case load. Aware that careworkers working in the community are on short term contracts, older persons highlighted how services change each time new contracts get issued:

… there were healthcare workers who used to be helpful but now they are no longer available ever since they changed groups, there is a new group now. Since then, we don’t see anyone bringing pills to older people, when they [older people] need pills, they have to visit the clinic to fetch their medication.

Older persons highlighted the need for public support, especially in contexts where families are unavailable to help. There was full appreciation and awareness that social workers were critical in getting support for older persons in the community.

## 7. Discussion: Underlying complexities and conflicts of care

The landscape of elderly care provision in post-apartheid South Africa is significantly shaped by the politics (financing) of caring which shapes the places (where care occurs and whether it is supported), and ultimately the relations of care. A strong policy focusses on ageing ‘in place’ places elderly care much more firmly in the family and community. Despite the policy focus on ‘ageing in place’, the financial de-prioritisation of community and familial care support services, demonstrates the state’s ongoing familialist approach to care provision. As our findings highlight, the state’s approach to care provision has a significant impact on older persons, caregivers, workers, families, and communities. Whilst the policy emphasises the place where care occurs in society, the examination of the budget and spending on older persons indicates the priority of care provision and support for older persons in the South African state’s agenda.

This article examined how spending on older persons has been heavily weighted towards the Older Person’s Grant. Whilst we recognise that the OPG is critical in providing much needed relief in households, there are glaring gaps in supporting direct care provision for elderly people, especially those living with disabilities. Furthermore, the reduction in spending on community and family-based care by 13 per cent over an 18-year period indicates not only the failure of the state to support ageing in place, but it indicates the state’s heightened retreat to pushing back care into the household.

With increasing care needs amongst older persons, there is a need for the South African state to bridge the gap between unsupported family care, limited frail care and uneven access to community care through service centres. In many countries across the region and Global South, elderly care is considered a family responsibility but is supported with a range of policies and public provision. For example, in the Seychelles, there is a Home Care Programme where paid home-based care is available and the programmes pay a cash benefit to cover the cost of a caregiver, at a rate of the minimum wage with full labour rights (WHO, 2017:16). In Mauritius, in addition to widely available day care centres and assistive devices and home health visits, caregivers of older people experiencing significant declines in capacity also receive a monthly allowance and some practical training (WHO, 2017:16). We recognise that comparisons from small island states with high income and older populations is not appropriate given that the priorities and starting points are different. However, an example from Brazil, a more comparable demographic, and economic context, shows us how other forms of supporting home-based care have been introduced. In Brazil the Programa Major Cuidado supports care-dependent older persons; paid caregivers are recruited from the community and provide families 10–20 h of support per week. It is offered as respite from care-work. There is training, the caregivers wear uniforms and act as a link between the families, and health and social assistance services (Lloyd-Sherlock et al., 2024). The inclusion of explicit home-based care programmes in long term care policies in the region and the Global South is still emerging and these are just some of the examples that are possible. Given the high cost and poor reach of residential care in South Africa and the high cost of hospital resources for managing disability related incidents (such as falls), investing more and prioritising community-based and home-based care is a key mechanism for supporting caregivers and older persons. Supporting older persons at home in providing services to those in need of assistance with instrumental and basic activities of daily living, currently the domain of Social Development, can reduce the demand for secondary and tertiary services (Solanki et al. 2021).

The findings demonstrate how an increase in the older persons population in South Africa, many of whom are living with comorbidities, and a decrease in spending on community – and family-based care deepens the centrality of households and women in families. The family and community are the central focus on elder care policy, and other forms of support are either not considered (such as geriatric home-based care) or considered too western (such as residential care) (Aboderin, 2004). Resonating with Hassim’s argument (2021:60) this ‘process (and persuasive discourse) delegitimises the possibilities of state-provided institutions of care.’ It also overlooks who performs care, under what conditions and with what consequences (Hassim, 2021:61; Sevenhuijsen et al., 2003). A dominant discourse prevails that older black South African persons have access to adequate care from family carers. Moreover, there are assumptions that traditions of care have their roots in cultural differences (Lloyd Sherlock, 2019:154) without any evidence about whether this resonates with older persons views or lived realities. In this paper we see that ‘tradition’ can be used to enforce spending restraint and neoliberal ‘self-reliance’ (by providing the OPG and GIA to take care of yourselves) reforms, all the while cutting the resources that are necessary to ensure care provision by family or community members. In the absence of budgetary commitments to supporting family and community care, many low-income black South African older persons and family caregivers are treated as unworthy of care from the state. The findings reinforce the reality of care as gendered, classed and racialised a point much overlooked by familialist approaches to family and welfare policy in South Africa.

The authors recognise that the family is viewed, by the state, as having the primary responsibility for supporting older persons. But the research shows that families are not always able to manage elder care and community care is not always available or supportive. Older persons, caregivers and careworkers at the community meetings called for more home-based support, more support with the GIA process and more age-friendly services. We argue that the state has a responsibility to uphold the principles and values that are set out in the Older Persons Act 2006 including a key provision that ‘older persons have the right to have their dignity respected and protected’ (s.35) and that given a key part of the Act is to shift the emphasis from institutional care to community care and the provision of home-based care, we would call for more support in this regard. Section 10 of the Act states that ‘an older person receiving community-based care and support services has, in addition to the rights contemplated in section 7, the right to (a) reside at home as long as possible (b) pursue opportunities for the full development of her potential and (c) benefit from family and community care and protection.’ We argue that the failure of the state to provide decent and widespread community care and to adequately support families in caring for older persons (through an increased uptake in the GIA) is contravening older persons rights to ‘age in place with dignity.’ Whilst the authors may regard the South African state’s ‘familialist by default’ approach as conservative and individualistic, we are concerned that the debate about the appropriate level of support has past given the increasing care needs of older persons. We argue that regardless of normative expectations of what families should do, care needs of older persons are increasing, and we need to support families now before there is a crisis, both economic, social and in the health sector.

The article describes the complexity of care provision for the elderly, with state, NPO organisations, communities and families all involved in providing care. As the state effectively reduces support for community-based services and NPOs, families and voluntary organisations are left to fill the gaps with very limited financial means. Moreover, in reviewing where financial support and state care provision is directed, we uncover how most support is either in the form of cash only (state grant) or targeted at the very frail and the very poor (residential care homes – which are still not enough for the very poor and very frail) as well as the relatively healthy and active (targeted at older persons who can access service centres). In this analysis we reveal that support for older persons, living in the community, who have medium to high care needs, are the gaping missing middle and least supported. Given the increase in direct care provision needed in communities, accessing, and supporting older persons who have medium to high care needs and providing direct care provision is the most urgent task for the state. The limited community-based care and support for family-based elderly care is creating tensions at both the community and family level. In reviewing the places of care provision, from the caregiver to the family, to the community and the wider financing of provision, we expose how conflictual care relations are being shaped at micro, meso and macro levels.

## Footnotes

1 In this paper we distinguish between unpaid family caregivers and paid careworkers. Family caregivers in the paper are referred to as unpaid caregivers and paid careworkers, such as social workers and home-based carers are referred to collectively as careworkers. We use the term home-based carers and social workers when we make distinctions between careworkers.

2 The phrase ‘ageing in place’ refers to staying in your own home as you get older.

3 The financial year 2006/2007 was chosen as a starting point for the mapping exercise as it coincides with the promulgation of the Older Persons Act of 2006. Data gaps in terms of allocations made to NPOs offering particular services limit detailed budget analysis in terms of the distribution of resources across different form of elder care in some provinces. The breakdown of services is focussed on DSD although we recognise that other departments such as Department of Health, may fund programmes or provide services to older persons, but older persons are not listed as a separate programme or budget programme, making it impossible to calculate spending outside of the DSD.
